# Lack of significant associations with early career performance suggest no link between the *DMRT3* “Gait Keeper” mutation and precocity in Coldblooded trotters

**DOI:** 10.1371/journal.pone.0177351

**Published:** 2017-05-10

**Authors:** Kim Jäderkvist Fegraeus, Chameli Lawrence, Katrine Petäjistö, Maria K. Johansson, Maja Wiklund, Christina Olsson, Leif Andersson, Lisa S Andersson, Knut H. Røed, Carl-Fredrik Ihler, Eric Strand, Gabriella Lindgren, Brandon D. Velie

**Affiliations:** 1 Department of Animal Breeding and Genetics, Swedish University of Agricultural Sciences, Uppsala, Sweden; 2 Department of Clinical Sciences, Swedish University of Agricultural Sciences, Uppsala, Sweden; 3 The Swedish Trotting Association, Bromma, Sweden; 4 Department of Medical Biochemistry and Microbiology, Uppsala University, Uppsala, Sweden; 5 Department of Veterinary Integrative Biosciences, Texas A&M University, College Station, Texas, United States of America; 6 Capilet Genetics AB, Västerås, Sweden; 7 Department of Basic Sciences and Aquatic Medicine, Faculty of Veterinary Medicine, Norwegian University of Life Sciences, Oslo, Norway; 8 Department of Companion Animal Clinical Sciences, Faculty of Veterinary Medicine, Norwegian University of Life Sciences, Oslo, Norway; University of Minnesota, UNITED STATES

## Abstract

The Swedish-Norwegian Coldblooded trotter (CBT) is a local breed in Sweden and Norway mainly used for harness racing. Previous studies have shown that a mutation from cytosine (C) to adenine (A) in the d*oublesex and mab-3 related transcription factor 3* (*DMRT3*) gene has a major impact on harness racing performance of different breeds. An association of the *DMRT3* mutation with early career performance has also been suggested. The aim of the current study was to investigate this proposed association in a randomly selected group of CBTs. 769 CBTs (485 raced, 284 unraced) were genotyped for the *DMRT3* mutation. The association with racing performance was investigated for 13 performance traits and three different age intervals: 3 years, 3 to 6 years, and 7 to 10 years of age, using the statistical software R. Each performance trait was analyzed for association with *DMRT3* using linear models. The results suggest no association of the *DMRT3* mutation with precocity (i.e. performance at 3 years of age). Only two traits (race time and number of disqualifications) were significantly different between the genotypes, with AA horses having the fastest times and CC horses having the highest number of disqualifications at 3 years of age. The frequency of the AA genotype was significantly lower in the raced CBT sample compared with the unraced sample and less than 50% of the AA horses participated in a race. For the age intervals 3 to 6 and 7 to 10 years the AA horses also failed to demonstrate significantly better performance than the other genotypes. Although suggested as the most favorable genotype for racing performance in Standardbreds and Finnhorses across all ages, the AA genotype does not appear to be associated with superior performance, early or late, in the racing career of CBTs.

## Introduction

Originating from North Swedish Draft horses and Norwegian Döle horses, the Coldblooded trotter (CBT) is a local breed found in both Sweden and Norway. While traditionally used in forest and agricultural work, the modern CBT is predominantly used in harness racing [1]. As a result, modern CBT breeders tend to focus on producing fast, durable horses with good temperament and conformation—as evidenced by the rapid improvement in harness racing performance over the last century [2,3]. However, despite heavy selection for harness racing merit within the breed, relatively little is known about the genes that underpin elite racing performance in CBT. Therefore, the discovery that a nonsense mutation in the d*oublesex and mab-3 related transcription factor 3* (*DMRT3*) gene has a major influence on harness racing performance was of great interest for the horse research community and the harness racing industry [4].

In Standardbreds, a breed predominantly used for harness racing, the *DMRT3* mutation has been shown to be of great importance for the ability to trot at high speed, with the majority of Standardbreds being homozygous for the mutation [4,5,6]. However, although Standardbreds and CBTs are bred and selected for the same purpose, the frequency of the *DMRT3* mutation was significantly lower in CBTs [5,6]. Moreover, the effect of the mutation was not as obvious in CBT. Most notably, homozygous A CBTs were significantly more successful (i.e. higher prize money earned) at 3 years of age, but as the horses matured the association weakened [6]. By contrast, studies of both Standardbreds and Finnhorses, a Finnish CBT, clearly demonstrated the superiority of AA horses for all traits regardless of age [6,7].

This juxtaposition regarding the superiority of AA horses is an important distinction to make given its potential for significantly altering the genomic architecture of the CBT breed. While not as extreme amongst CBT breeders compared to Thoroughbreds or Standardbreds, the desire for a quick return on a horse is common across all racing breeds. The nature of the industry, be it gallop or harness racing, tends to favor precocious horses that can not only start competing, but also win, at a young age. The possible association between a single gene and precocity could easily push the selection of CBT in one direction, reducing the genetic variation of an already limited population. With this in mind, the aim of the current study was to investigate the suggested precociousness of AA horses, as well as the association between the *DMRT3* mutation and racing performance at older ages, in a randomly selected horse material.

## Materials and methods

### Sample collection

To ensure at least 475 raced horses (equivalent to five 96-well plates) and to accurately reflect the proportion of raced and unraced horses in the population, a sample of 1000 registered Swedish/Norwegian CBTs born between 2000–2009 were randomly selected from the database of the Swedish- and the Norwegian Trotting Association, using the software program for statistical computing R [8]. Hair and blood samples were obtained for the first 770 horses listed (485 raced, 285 unraced) from the Animal Genetics Laboratory at the Swedish University of Agricultural Sciences, Uppsala, Sweden and the Norwegian University of Life Sciences, Oslo, Norway. The study was approved by the Ethics Committee for Animal Experiments in Uppsala, Sweden (Number C121/14).

### DNA extraction and SNP genotyping

DNA was isolated from hair roots using a standardized procedure. Five hair roots were cut into separate wells in 96-well plate. A solution of 100 μl Chelex (5%) and 7 μl proteinase K was added to each well. The 96-well plates were incubated at 56°C for 60 minutes and 95°C for 10 minutes, to inactivate proteinase K. The DNA from blood samples was extracted from 350 μl blood using the Qiasymphony instrument (Qiagen, Hilden, Germany).

The SNP genotyping of the *DMRT3_Ser301STOP* marker (ECA23:22,999,655) was performed using the StepOnePlus Real-Time PCR System (Life Technologies) with custom- designed TaqMan SNP Genotyping Assays (Applied Biosystems).

### Performance data

Performance data for the years 2003–2015 was provided by the Swedish Trotting Association. To investigate the effect of *DMRT3* at different ages, three different age intervals were defined; 3 years, 3 to 6 years, and 7 to 10 years of age. The age interval 3 to 6 years was chosen as the genetic evaluation of CBTs is based on performance results and racing status between the ages 3 to 6 years [9]. The following performance traits were analyzed:

*Rankings*: The number of victories was calculated as the total number of times a horse finished a race in first place. The number of placings was calculated as the total number of times a horse finished a race in first, second or third place. The number of unplaced was calculated as the total number of times when a horse finished a race in fourth place or lower.
*Racing times*: Two different start methods were included in the study for the race times: auto- and voltstart [1]. The best racing times for each horse were defined as the lowest time (in seconds) per kilometer, for each start method.
*Earnings*: Earnings were calculated as the amount of prize money a horse had earned at each age interval. The majority of the earnings provided were in Swedish currency (SEK), but the earnings for Norwegian trotters were in Norwegian currency (NOK). In order to set all earnings to Swedish currency an average exchange rate was calculated (μ = 0.95) for the years 2003–2015 and multiplied with the Norwegian earnings [10]. Earnings per start were calculated as the amount of prize money earned per start.
*Disqualifications*: The number of disqualifications was calculated as the total number of times a horse was disqualified as a result of galloping or pacing during a race.
*Estimated breeding values (EBVs)*: Estimated breeding values are based on the individual’s results (40% racing status and 60% earnings for CBTs), as well as the relatives’ results. EBVs are adjusted for the fixed effects of sex and age, and updated annually [9]. In this study, the obtained EBVs were from 2015.

### Statistical analysis

All statistical analyses were performed using R [8]. Hardy Weinberg equilibrium (HWE) was evaluated using the R package “SNPassoc” [11]. Summary statistics for each performance traits were calculated based on raw values. Each trait was tested for normality using the Shapiro-Francia test. To get normally distributed values, the earnings and best racing times were transformed according to a previously published formula: ln(earnings + 1 000) and ln(racing time– 68.2), respectively [12]. All other non-normally distributed traits were log_10_-transformed. Horses with no time records using autostart or voltstart for respectively age interval were excluded from all analyses concerning the corresponding starting method.

In order to determine the association of the *DMRT3* genotype with performance, each trait was analyzed using linear models. All models, except for those used for analysis of EBVs, included fixed effects of sex, birthdate and country of registration. Covariates such as number of starts and earnings were included when applicable. A single marker association analysis was performed using ANOVA. If the result was significant (*P*≤0.05), a multiple comparison test was performed using Tukey’s HSD-test [13].

The difference in *DMRT3* frequency between raced and unraced horses was analyzed using a linear model including the fixed effects of sex, age and country of registration. The association analyses were performed using ANOVA, followed by Tukey’s HSD-test.

## Results

### Population description

**Fig 1:**
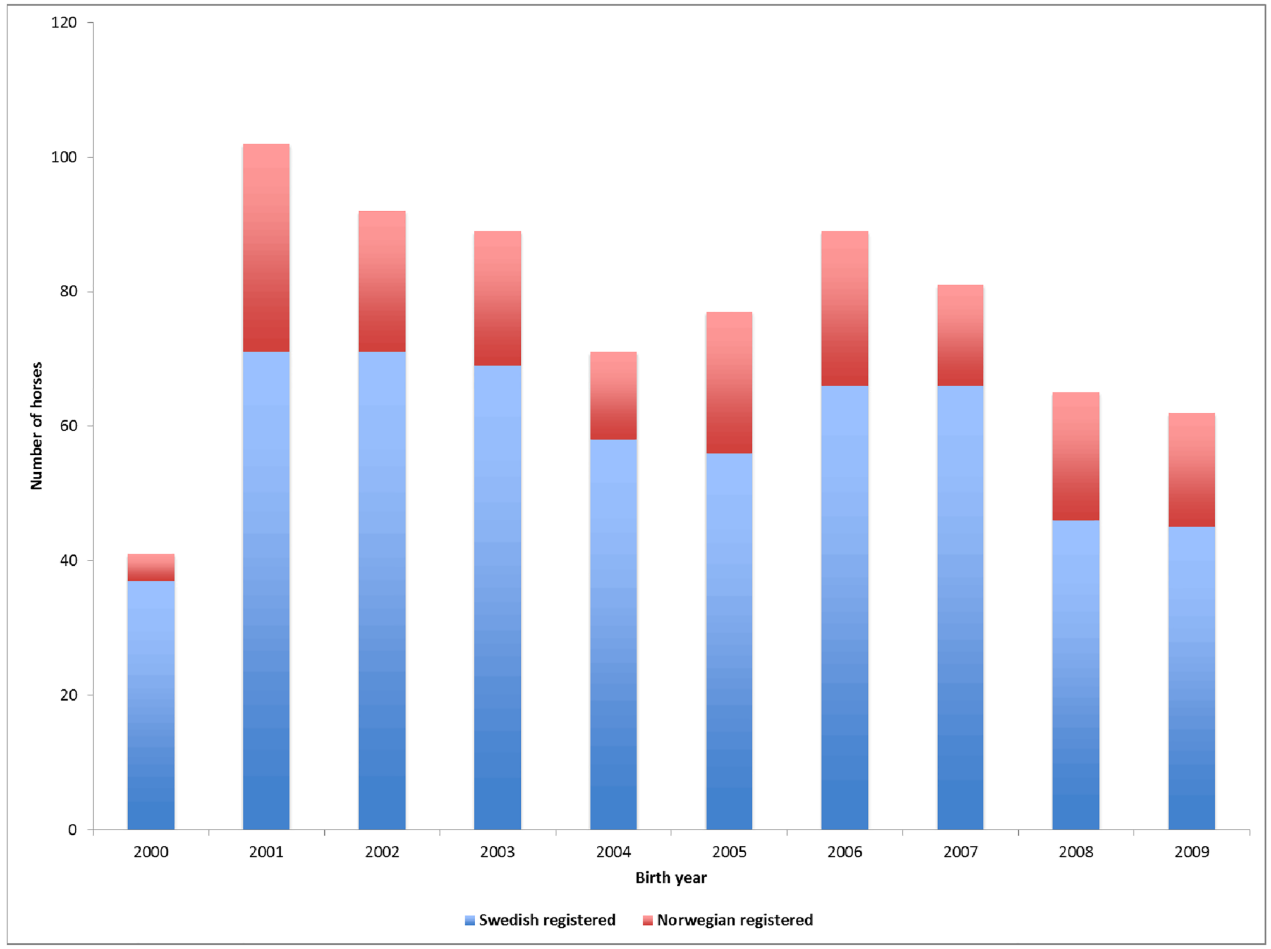
Distribution of birth year (n = 769).

A total of 769 horses were successfully genotyped for the *DMRT3* SNP. The distribution of birth year and country of registration is presented in Fig 1.

**Fig 2:**
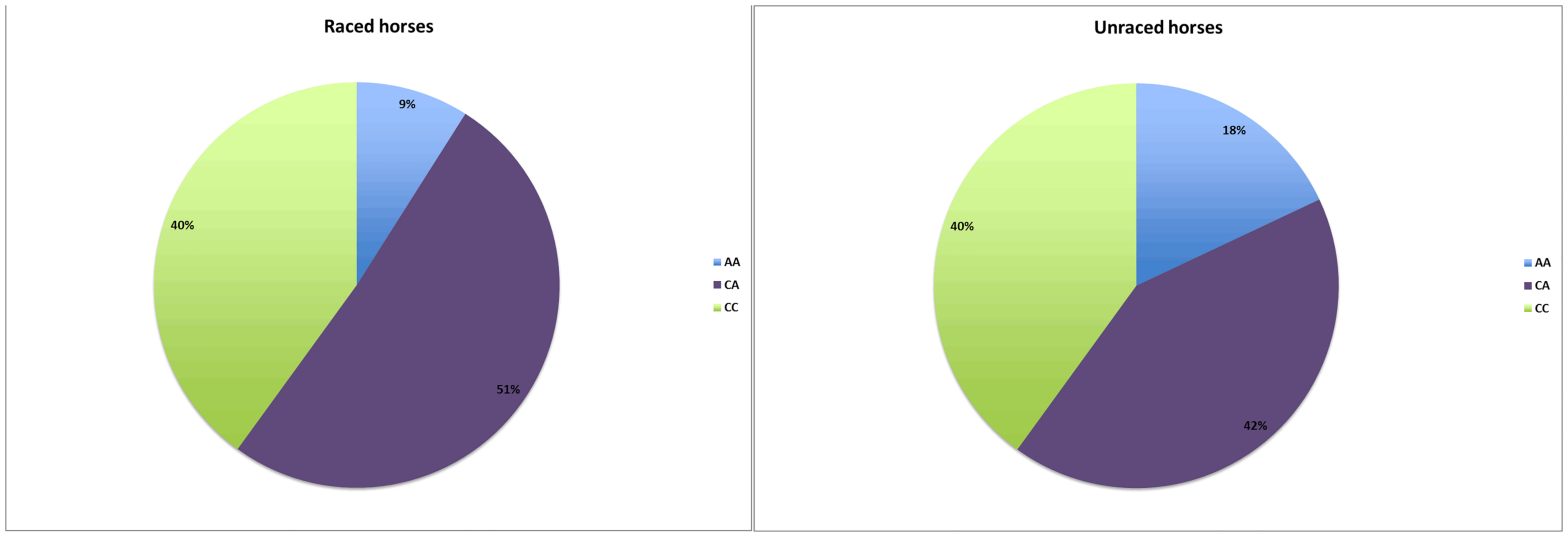
*DMRT3* genotype frequency in raced and unraced horses.

**Fig 3:**
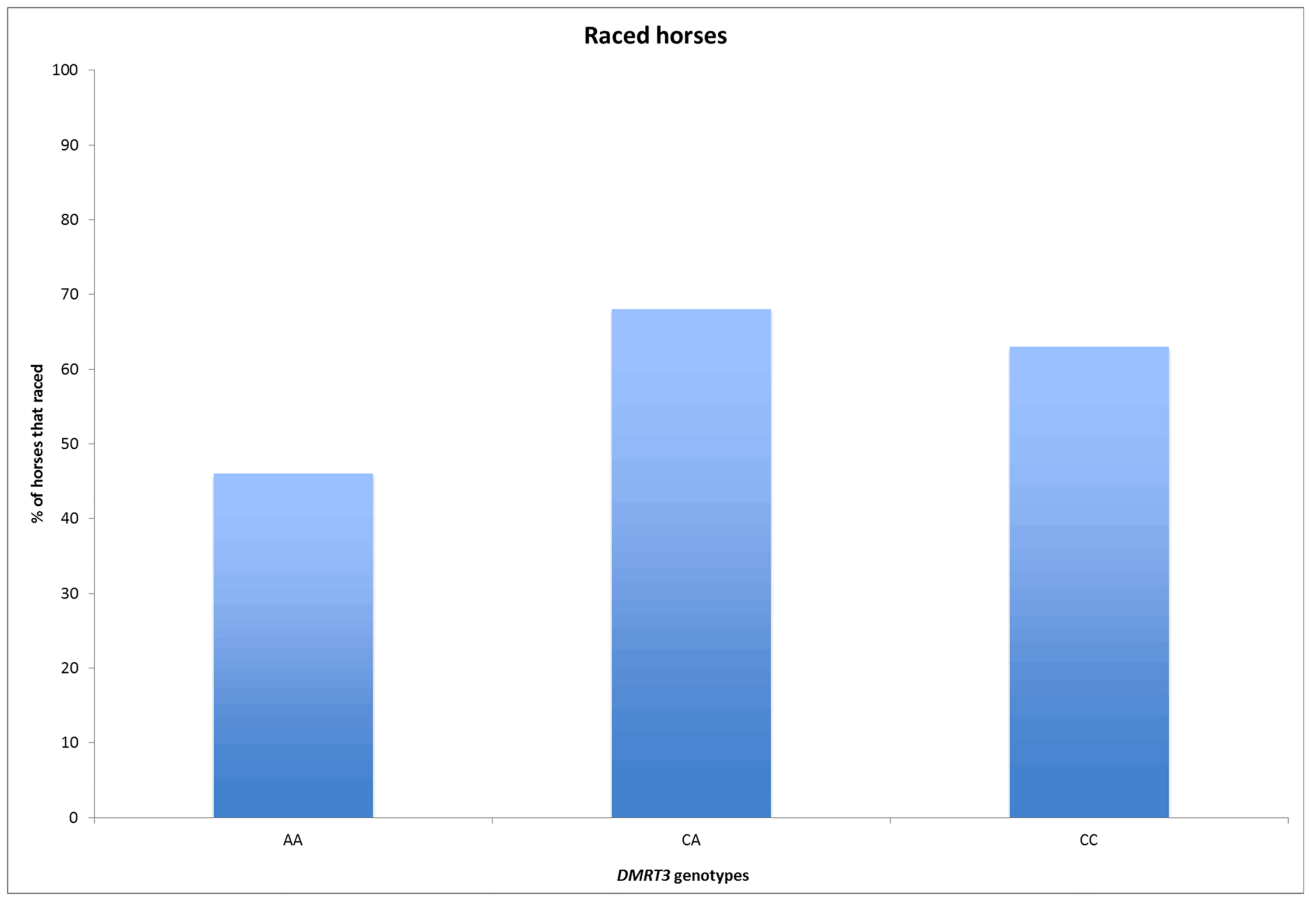
Percentage of raced horses for each *DMRT3* genotype.

The total sample consisted of 90 stallions, 332 geldings and 347 mares from 128 sires, with an average of 6 offspring per sire. The number of offspring per sire ranged from 1 to 72. The frequency of the AA genotype was significantly higher (*P* = 0.05) in the group of unraced horses than in the group of raced horses (Fig 2). The genotypes for the raced and unraced horses deviated significantly from HWE (*P* = 0.004 respectively *P* = 0.05), while the genotypes for all horses combined (n = 769) did not deviate from HWE (*P* = 0.35). Fifty-five percent of the raced horses started their first race at 3 years of age and there were no significant differences between the genotypes (Table 1). The average age for the first race was 3.8 years and it did not differ between the genotypes (Table 1). The proportions of raced horses for each genotype are presented in Fig 3. The descriptive performance results are presented in S1 Table.

**Table 1:** The percentage of raced Coldblooded trotters that started their first race at 3 years of age, and the average age for the first start, presented for each *DMRT3* genotype.

	AA	CA	CC	*P*-value^1^
Started at 3 years of age (%)	56	57	52	0.56
Average age for first race (years)	3.7	3.7	3.8	0.44

^1^A Kruskal-Wallis test was performed in R

### Performance analysis

The fixed effects and covariates used in the linear models for each performance trait, including *P*-values, are presented in S2 Table. While most of the performance analyses included all 485 raced horses, the analyses of disqualifications included only 350 horses. The remaining (n = 135) horses were excluded due to insufficient availability of information pertaining to disqualifications.

At 3 years of age, there were two significant differences between the genotypes; the race time where the AA horses were significantly faster than the CC horses and the disqualifications where the CC horses had the highest number (Table 2). For the age interval 3 to 6 years, the CA horses performed significantly better than the CC horses for the majority of the traits. Also, the AA horses had significantly more placings and fewer disqualifications than the CC horses, but the lowest earnings (Table 3). At 7 to 10 years of age, the CA and CC horses had significantly more starts than the AA horses. The AA horses also had the lowest number of placings and earnings. Additionally, the heterozygous horses had significantly better race times compared to the other genotypes. The CC horses had the highest number of disqualifications (Table 4). For a complete summary of the performance statistics for the different age classes, see S3–S5 Tables. The average EBV of the raced horses was 108.5 and it did not differ between genotypes (*P*>0.05).

**Table 2:** Mean and median racing performance results for Coldblooded trotters at 3 years of age according to *DMRT3* genotype (n = 268).

Trait^1^	AA (n = 5–24)			CA (n = 35–143)			CC (n = 23–101)			*P*-value^2^		
	Mean	Median	SE	Mean	Median	SE	Mean	Median	SE	AA/CA	CA/CC	AA/CC
No. of starts	6.3	6.5	0.8	6.0	5.0	0.4	5.8	5.0	0.4	0.84	0.66	0.55
No. of victories	0.8	1.0	0.2	0.9	0	0.1	0.7	0	0.1	0.77	0.65	0.47
Victories (freq.)	0.088	0.079	0.019	0.101	0	0.015	0.083	0	0.015	0.99	0.66	0.94
No. of placings (1–3)	2.1	2.0	0.4	2.3	1	0.3	1.9	1.0	0.2	0.94	0.32	0.49
Placings (1–3) (freq.)	0.245	0.261	0.047	0.291	0.250	0.025	0.236	0.200	0.025	0.80	0.30	0.97
No. of unplaced	3.0	3.0	0.43	2.6	2.0	0	2.4	2.0	0.2	0.27	0.97	0.24
Unplaced (freq.)	0.563	0.506	0.07	0.481	0.500	0	0.497	0.500	0.033	0.48	0.91	0.65
No. of disqualifications^3^	0.5	0	0.2	0.6	0	0.1	1.1	1.0	0.2	0.86	**0.02**	0.09
Disqualifications (freq.)^3^	0.126	0	0.064	0.159	0	0.028	0.223	0.092	0.040	0.89	0.27	0.37
Earnings (SEK)	47 960	25 320	12 493	61 830	15 160	13 646	35 090	15 380	5 314	0.49	0.23	0.11
Earnings per start (SEK)	6 128	3 278	1 651	6 136	3 480	900	4 358	2 579	563	0.81	0.75	0.57
Race time autostart (sec/km)^4^	92.0	92.2	0.5	91.5	91.4	0.2	92.9	92.4	0.3	0.83	0.06	0.76
Race time voltstart (sec/km)^5^	94.6	94.0	0.9	95.8	95.1	0.4	96.0	95.4	0.4	0.09	0.82	**0.05**

^1^ Transformed values were used for the analysis: log_10_, ln(earnings + 1 000) and ln(race time—68.2)

^2^ A multiple comparison test was performed using Tukey´s HSD test. Significant results (*P*≤0.05) in bold

^3^ n = 176

^4^ n = 63

^5^ n = 245

**Table 3:** Mean and median racing performance results for Coldblooded trotters at 3 to 6 years of age according to *DMRT3* genotype (n = 472).

Trait^1^	AA (n = 26–41)			CA (n = 149–243)			CC (n = 99–188)			*P*-value^2^		
	Mean	Median	SE	Mean	Median	SE	Mean	Median	SE	AA/CA	CA/CC	AA/CC
No. of starts	23.4	18.0	2.7	26.4	23.0	1.3	23.0	18.0	1.3	0.27	**0.002**	0.89
No. of victories	2.9	2.0	0.5	3.3	2.0	0.3	2.6	1.0	0.3	0.95	**0.007**	0.11
Victories (freq.)	0.126	0.111	0.020	0.111	0.080	0.008	0.088	0.054	0.008	0.68	0.11	0.13
No. of placings (1–3)	8.2	8.0	1.0	8.8	6.0	0.6	7.0	4.0	0.6	0.93	**<0.001**	**0.007**
Placings (1–3) (freq.)	0.323	0.329	0.031	0.298	0.286	0.013	0.243	0.225	0.014	0.70	**0.005**	**0.03**
No. of unplaced	11.0	7.0	1.5	12.0	11.0	0.6	11.0	9.0	0.6	0.31	**0.004**	0.93
Unplaced (freq.)	0.499	0.471	0.034	0.480	0.462	0.014	0.490	0.500	0.016	0.84	0.88	0.96
No. of disqualifications^3^	1.8	1.0	0.4	2.7	2.0	0.2	3.5	3.0	0.3	0.24	**0.04**	**0.007**
Disqualifications (freq.)^3^	0.111	0.043	0.024	0.142	0.100	0.013	0.229	0.188	0.019	0.65	**<0.001**	**0.003**
Earnings (SEK)	128 100	98 500	20 625	179 300	72 500	18 543	133 200	54 370	17 154	0.72	**<0.001**	**0.03**
Earnings/start (SEK)	4 963	3 895	594	5 417	3 781	428	4 178	2 873	355	1.00	**0.007**	0.19
Race time autostart (sec/km)^4^	89.1	88.9	0.4	89.5	88.8	0.5	89.9	89.8	0.3	0.76	**0.002**	0.39
Race time voltstart (sec/km)^5^	91.1	90.0	0.7	91.2	90.7	0.3	91.9	91.8	0.3	0.86	**0.005**	0.06

^1^ Transformed values were used for the analysis: log_10_, ln(earnings + 1 000) and ln(race time—68.2)

^2^ A multiple comparison test was performed using Tukey´s HSD test. Significant results (*P*≤0.05) in bold

^3^ n = 337

^4^ n = 275

^5^ n = 453

**Table 4:** Mean and median racing performance results for Coldblooded trotters at 7 to 10 years of age according to *DMRT3* genotype (n = 182).

Trait^1^	AA (n = 14–18)			CA (n = 71–98)			CC (n = 44–66)			*P*-value^2^		
	Mean	Median	SE	Mean	Median	SE	Mean	Median	SE	AA/CA	CA/CC	AA/CC
No. of starts	16.2	13.0	4.6	25.3	18.0	2.3	23.5	15.0	2.7	**0.004**	0.94	**0.01**
No. of victories	1.2	0	0.6	2.5	1.0	0.5	1.8	1.0	0.3	0.13	0.62	0.39
Victories (freq.)	0.048	0	0.016	0.074	0.043	0.010	0.067	0.046	0.011	0.51	0.89	0.71
No. of placings (1–3)	3.8	1.5	1.4	6.9	3.0	0.9	5.5	3.0	0.8	**<0.001**	0.38	**0.02**
Placings (1–3) (freq.)	0.174	0.141	0.042	0.211	0.200	0.017	0.186	0.189	0.017	0.59	0.66	0.90
No. of unplaced	10.0	6.5	3.0	14.0	10.5	1.0	12.0	9.0	1.4	**0.005**	0.10	0.16
Unplaced (freq.)	0.630	0.667	0	0.583	0.560	0	0.531	0.526	0.029	0.77	0.41	0.33
No. of disqualifications^3^	1.5	1.0	0.4	2.6	1.0	0.5	4.1	3.0	0.6	0.36	**0.02**	**0.006**
Disqualifications (freq.)^3^	0.120	0.056	0.041	0.178	0.101	0.032	0.269	0.233	0.035	0.68	0.12	0.09
Earnings (SEK)	62 120	23 020	26 265	168 200	53 900	33 435	114 000	40 140	28 748	**0.004**	0.20	0.10
Earnings per start (SEK)	2 903	2 402	556	4 371	3 283	513	3 328	2 482	465	1.00	0.71	0.85
Race time autostart (sec/km)^4^	89.7	89.6	0.6	87.7	87.7	0.3	89.1	88.8	0.4	**0.006**	**0.007**	0.54
Race time voltstart (sec/km)^5^	90.3	90.5	0.8	89.2	89.0	0.4	90.1	89.5	0.5	0.15	**0.05**	0.93

^1^ Transformed values were used for the analysis: log_10_, ln(earnings + 1 000) and ln(race time—68.2)

^2^ A multiple comparison test was performed using Tukey´s HSD test. Significant results (*P*≤0.05) in bold

^3^ n = 122

^4^ n = 129

^5^ n = 168

## Discussion

Although a previous study indicated a favorable association between the AA genotype for *DMRT3* and superior performance in young CBTs, the current study provides no evidence to support such an association with *DMRT3* [6]. The lack of concordance between the studies may be due to the larger and randomly selected horse material used in the current study. Only two significant differences (race time for voltstart and number of disqualifications) were found between the genotypes and traits associated with precocity (i.e. performance at 3 years of age) (Table 2). Additionally, the proportion of AA horses that raced for the first time at 3 years of age and the average age for the first race did not differ significantly from the other genotypes. If AA horses were truly more precocious, a clear difference would be apparent, with a greater proportion of AA horses racing at 3 years of age. As observed in Thoroughbreds where one variant of the *Myostatin* gene is associated with early performance and sprinting ability [14], a positive association of a specific genotype with precocity would likely result in a push towards that genotype in the population. The fact that the majority of the CBTs are heterozygous for the *DMRT3* mutation, despite a desire for precocious horses, strongly suggests no significant association between *DMRT3* and precocity.

Perhaps a more interesting observation is the rather low proportion of raced AA horses (Figs 2 and 3). As the ability to race is the most important factor for a successful racehorse, the large proportion of unraced AA horses suggests a possible unfavorable association of the genotype with racing performance. While the genotypes for the whole population did not deviate from HWE, the genotypes for the raced and unraced horses did deviate from HWE when analyzed separately. Given *DMRT3*s association with performance this may be an indicator of the ongoing selection for racing performance in the CBTs. The AA horses that made it to the racetrack performed well up to six years of age, as indicated by high median values for earnings and victories (Tables 2 and 3). Although successful at young ages, the AA horses did not perform as well for the older ages, where the median values for earnings and victories were the lowest of the three genotypes (Table 4). Also, it is worth noticing that the AA horses had the lowest number of starts for all ages except 3 years of age. This has also been seen in Finnhorses, where the AA horses, despite the superior effect of the homozygous genotype, had the lowest number of starts for all ages above four years of age [7]. Although the low number of starts for AA Finnhorses may be explained by their superior performance, as successful racehorses tend to compete less often, the low number of starts for both AA Finnhorses and CBTs may also indicate an association between the AA genotype and poor durability.

Albeit still uncharted and possibly controversial, the disparate effect of the AA genotype on performance in CBTs is potentially influenced by an increased preference of AA CBTs for the gait pace [6]. Pacing in a race will lead to disqualification and therefore horses are prevented from pacing by the use of different weights and shoes to balance the horse. Heavy balancing of young horses could potentially increase the risk for injuries, which in turn may have a detrimental impact on performance. Although a potential reason for the low frequency of the AA genotype in the CBT population, not all AA CBTs have a propensity for pace. Some AA horses possess a conformation that makes them more prone to pace, but not all AA horses naturally pace, as demonstrated in Icelandic horses [15]. Perhaps a similar occurrence is observed in CBTs.

The CC CBTs in the current study were the least successful prior to six years of age, but their performance improved as they matured (Table 4). CC CBTs reportedly display an inferior trotting technique compared to the other genotypes [6], which is often indicative of a horse that prefers to gallop rather than extend the trot when prompted for an increase in speed. This is supported by the high frequency of the CC genotype in breeds traditionally used for gallop races or recreation as well as the high numbers of disqualifications for the CC horses in the current study [4,5,6]. Moreover, previous studies have demonstrated an unfavorable association between the CC genotype and harness racing performance in other breeds [6,7,16]. While the exact reasons for this are unknown, it is possible that this poor performance is rooted in a propensity to gallop. Despite this possible gait preference in CC CBTs, the frequency of the CC genotype is still relatively high in the CBT population [5,6]. While CC CBTs may posses a conformation better suited for galloping, the current study suggests that with time these horses can develop proper trotting technique and eventually become solid racehorses.

## Conclusion

The *DMRT3* mutation does not appear to influence precocity in CBTs. However, the current study suggests an association of *DMRT3* genotype and the ability to start racing. As the effect of the *DMRT3* mutation in CBTs appears to be disparate from the effect seen in other harness racing breeds, additional studies on CBTs would be beneficial to fully understand the influence of the gene on harness racing performance.

## Supporting information

S1 Table Summary statistics of performance traits. (DOCX)

S2 Table *P*-values for fixed effects and covariates used in the models for estimation of performance, at 3 years, 3 to 6 years, and 7 to 10 years of age. (DOCX)

S3 Table Descriptive performance results for 3 years of age stratified by *DMRT3* genotype (n = 268). (DOCX)

S4 Table Descriptive performance results for 3 to 6 years of age stratified by *DMRT3* genotype (n = 472). (DOCX)

S5 Table Descriptive performance results for 7 to 10 years of age, stratified by *DMRT3* genotype (n = 182). (DOCX)
