# Supplementary material for: Lack of significant associations with early career performance suggest no link between the DMRT3 “Gait Keeper” mutation and precocity in Coldblooded trotters
Source: PLoS One. 2017 May 10;12(5):e0177351. doi: 10.1371/journal.pone.0177351 (PMC5425215; doi:10.1371/journal.pone.0177351)
Supplement: S1 Table — (DOCX) [file pone.0177351.s001.docx]

**S1 Table. Summary statistics of performance traits**

| **Performance trait** | **Min** | **1st Quantile** | **Median** | **Mean** | **3rd Quantile** | **Max** |
| --- | --- | --- | --- | --- | --- | --- |
| **Raced horses (n=485)** |  |  |  |  |  |  |
| EBV^1^ | 95.0 | 107.0 | 112.0 | 111.7 | 117.0 | 126.0 |
| No. of starts | 1.0 | 13.0 | 28.0 | 38.0 | 53.0 | 222.0 |
| No. of victories | 0 | 0 | 2.0 | 4.0 | 5.0 | 53.0 |
| No. of placings (1-3) | 0 | 2.0 | 7.0 | 11.3 | 15.0 | 101.0 |
| No. of unplaced | 0 | 5.0 | 14.0 | 19.0 | 27.0 | 133.0 |
| No.of disqualifications^2^ | 0 | 1.0 | 3.0 | 4.4 | 6.0 | 46.0 |
| Earnings (SEK) | 0 | 31 250 | 97 620 | 229 800 | 250 500 | 3 189 000 |
| Earnings per start (SEK) | 0 | 1 979 | 3 474 | 4 758 | 5 510 | 54 220 |
| Race time autostart (sec/km)^3^ | 80.1 | 86.5 | 88.6 | 88.7 | 90.9 | 99.3 |
| Race time voltstart (sec/km)^4^ | 81.4 | 88.1 | 90.2 | 90.6 | 93.0 | 106.9 |
|  |  |  |  |  |  |  |
| **Population (n=769)** |  |  |  |  |  |  |
| EBV^1^ | 89.0 | 104.0 | 108.0 | 109.0 | 114.0 | 126.0 |
| No. of starts | 0 | 0 | 10.0 | 24.0 | 38.0 | 222.0 |
| No. of victories | 0 | 0 | 0 | 3.0 | 3.0 | 53.0 |
| No. of placings (1-3) | 0 | 0 | 2.0 | 7.0 | 10.0 | 101.0 |
| No. of unplaced | 0 | 0 | 4.0 | 12.0 | 18.0 | 133.0 |
| Earnings (SEK)^5^ | 0 | 0 | 24 300 | 145 000 | 134 200 | 3 189 000 |

^1^ Estimated Breeding Value

^2^ n= 350

^3^ n= 319

^4^ n= 467

^5^ Unraced horses were given a value of -1 for earnings
