# Supplementary material for: Lack of significant associations with early career performance suggest no link between the DMRT3 “Gait Keeper” mutation and precocity in Coldblooded trotters
Source: PLoS One. 2017 May 10;12(5):e0177351. doi: 10.1371/journal.pone.0177351 (PMC5425215; doi:10.1371/journal.pone.0177351)
Supplement: S2 Table — (DOCX) [file pone.0177351.s002.docx]

**S2 Table. *P*-values for fixed effects and covariates used in the models for estimation of performance, at 3 years, 3 to 6 years, and 7 to 10 years of age**
