# Supplementary material for: Lack of significant associations with early career performance suggest no link between the DMRT3 “Gait Keeper” mutation and precocity in Coldblooded trotters
Source: PLoS One. 2017 May 10;12(5):e0177351. doi: 10.1371/journal.pone.0177351 (PMC5425215; doi:10.1371/journal.pone.0177351)
Supplement: S5 Table — (DOCX) [file pone.0177351.s005.docx]

**S5 Table. Descriptive performance results for 7 to 10 years of age, stratified by *DMRT3* genotype (n=182)**

^^

^1^ n= 122

^2^ n= 129

^3^ n= 168
